# Enhancing Rumen Fermentation and Bacteria Community in Sika Deer (*Cervus nippon*) through Varying Levels of Dragon Fruit Peel Polyphenolic Extract: An In Vitro Study

**DOI:** 10.3390/ani14081139

**Published:** 2024-04-09

**Authors:** Chao Ban, Xingzhou Tian, Qi Lu, Pipat Lounglawan, Guilan Wen

**Affiliations:** 1 School of Animal Technology and Innovation, Institute of Agricultural Technology, Suranaree University of Technology, Nakhon Ratchasima 30000, Thailand; ban_chao@outlook.com; 2 College of Animal Science, Guizhou University, Guiyang 550025, Chinaqlu@gzu.edu.cn (Q.L.); 3 Key Laboratory of Animal Genetics, Breeding and Reproduction in the Plateau Mountainous Region, Ministry of Education, College of Animal Science, Guizhou University, Guiyang 550025, China

**Keywords:** dragon fruit peel, in vitro gas production, polyphenolic extract, rumen fermentation, sika deer, 16S rRNA sequencing

## Abstract

**Simple Summary:**

Dragon fruit peel polyphenolic extract is a potential ruminal modulator endorsed by consumers for its inherent safety and acceptability. This in vitro study demonstrated that dragon fruit peel polyphenolic extract increased the concentration of volatile fatty acids in the rumen, reduced gas production, and enhanced the rumen bacterial abundances. These findings suggest that dragon fruit peel polyphenol extract has the potential to improve ruminal fermentation of sika deer and provides a theoretical basis for dragon fruit peel polyphenol extract to be used as a functional feed additive for Sika deer.

**Abstract:**

The aim of this study is to investigate the effect of dragon fruit peel polyphenolic extract (DFPE) on gas production, rumen fermentation, and bacterial communities in sika deer using an in vitro technique. Three treatments with different DFPE levels (DFPE0, base diet; DFPE5, base diet + 5 mg/g DFPE; DFPE10, base diet + 10 mg/g DFPE, respectively; n = 6) were implemented. The phenolic composition of DFPE, gas production (GP), ammonia nitrogen (NH_3_-N), volatile fatty acid (VFA), and bacteria communities was evaluated after 24 h of incubation. The results showed that GP and NH_3_-N were reduced by DFPE supplementation. Total VFA, isovaleric acid, and valeric acid were increased (*p* < 0.05) by the addition of DFPE. No changes (*p* > 0.05) were observed in pH, acetic acid, propionic acid, isobutyric acid, butyric acid, and the ratio of acetic acid to propionic acid. Additionally, the alpha indexes, including Sobs, Shannon, and Ace, were increased by DFPE supplementation. Moreover, at the phylum level, DFPE supplementation increased (*p* = 0.01) *Bacteroidota* but reduced (*p* < 0.01) *Firmicutes*. At the genus level, compared to DFPE0, the DFPE10 had increased relative abundances of *Rikenellaceae_RC9_gut_group* (*p* < 0.01), *norank_f_Muribaculaceae* (*p* = 0.01), *Lachnospiraceae_NK3A20_group* (*p* < 0.01), *Christensenellaceae_R-7_group* (*p* < 0.01), and *NK4A214_group* (*p* < 0.01), decreased relative abundances of *Streptococcus* (*p* < 0.01), *Oribacterium* (*p* = 0.01), and *Enterococcus* (*p* < 0.01). Compared to DFPE0, DFPE5 had no change (*p* > 0.05) in all bacteria at the genus level except for decreased relative abundance of *Enterococcus* (*p* < 0.01). These results indicated that DFPE may be able to be used as a feed additive to enhance fermentation parameters and improve ruminal bacteria communities in Sika deer.

## 1. Introduction

The structure of a ruminal microbial community is influenced by various factors, including health status, physiological status (e.g., pregnancy, lactation) [1], individual variation, and diet composition. Of these, diet composition exerts a dominant influence over the composition of ruminal microbiota [2,3]. In addition to diet, plant secondary metabolites, especially phenolic compounds, play a crucial role in the formation of ruminal microbial ecosystems [4]. For instance, naringin, which is a flavonoid (a subclass of polyphenols) compound commonly found in fruit peel, has been shown to reduce methane production to improve feed efficiency and increase the ruminal butyrate ratio while altering the composition of ruminal bacteria [5]. Tian et al. [6] found that polyphenols-rich sorghum stalks, which contain various flavonoids, including naringin, could reduce ruminal gas production and increase propionic acid proportions. Thus, enhancing rumen fermentation by utilizing polyphenols represents an effective approach to safeguarding the health of ruminant animals, increasing animal product yield and improving product quality.

Dragon fruit (*Hylocereus polyrhizus*) peel polyphenolic extract can be considered an antioxidant source that is non-toxic and biologically safe, serving as the functional feed additive that has the potential to improve rumen fermentation. Previous research has shown that dietary dragon fruit peel could increase microbial protein supply in Holstein crossbred bulls [7]. Furthermore, dragon fruit peel could increase the content of volatile fatty acids and reduce methane production [8].

Sika deer (*Cervus nippon*) is a special livestock that is widely distributed in Asian countries, including China, Vietnam, Korea, and Japan [9]. The velvet antler of Sika deer serves as the primary product, which is an exceedingly coveted natural resource with substantial market potential [10]. Understanding the bacterial ecology within the rumen of Sika deer is crucial for optimizing nutrient digestion efficiency and boosting velvet antler productivity [11]. However, there is a scarcity of research on the utilization of phenolic substances in Sika deer, particularly in relation to their impact on rumen microorganisms, which remains largely unexplored. Therefore, we hypothesized that supplementing dragon fruit peel polyphenolic extract could enhance rumen fermentation and the microbial population. The current study aimed to investigate dragon fruit peel polyphenolic extract on gas production, ruminal fermentation characteristics, and bacteria communities in Sika deer using an in vitro technique.

## 2. Materials and Methods

### 2.1. Animal Care

The animal study underwent a thorough review, ensuring that all procedures related to the care and treatment of experimental animals were carefully examined and assessed. Reviewing department: Animal Welfare and Experimental Animal Ethics of Guizhou University (EAE-GZU-2024-E001), Guizhou, China.

### 2.2. Experimental Design

Three treatments were administered involving the supplementation of polyphenols from dragon fruit peel extract (DFPE was commercial extract, it was purchased from Ningshan Guosheng Biotechnology Co., Ltd., Ankang, China). These treatments were distinguished by varying levels of dragon fruit peel extract: 0, 5, and 10 mg/g of fermentation substrate based on dry matter (DM), respectively. Each treatment was 6 repetitions. The in vitro fermentation substrate composition is shown in Table 1.

### 2.3. Simulating Rumen Fermentation Processes In Vitro

Two male Sika deer (BW = 78.41 ± 6.82 kg, mean ± SD) at 2 years old were selected from Guizhou Shengchangyao Ecological Agriculture Comprehensive Development Co., Ltd. (Bijie, China) as the donors of rumen fluid.

All Sika deer were housed in pens with free access to fresh drinking water and were fed twice a day at 8:00 am and 4:00 pm. The feed had a concentrate-to-roughage ratio of 30:70, and the dietary nutrient requirements were sourced from the National Research Council [12]. The roughage was corn stalk, and the composition of concentrate was (based on DM) 21.6% corn, 16.6% rice bran, 38.8% soybean meal, wheat bran 20.5%, 1% salt, 1% calcium bicarbonate, and 0.5% premix (the premix consists of vitamin premix 700 IU/g, Fe 10 mg/g, Zn 3 mg/g, Cu 0.75 mg/g, Mn 3 mg/g, Se 0.01 mg/g, Co 0.02 mg/g).

The rumen fluid was collected after 12 h of fasting, and the deer were anesthetized with 2 mL xylazine hydrochloride injection (Jilin Huamu Animal Health Products Co., Ltd., Changchun, China) using an anesthetic gun. Rumen fluids were obtained via mouth by a stomach tube connected to a vacuum pump. The rumen fluids were immediately placed into a prefilled CO_2_ thermos in equal proportions and transferred to the laboratory. Subsequently, rumen fluids were filtered through four layers of gauze while being continuously flushed with CO_2_. According to the method of Menke and Steingass [13], the artificial saliva was configured as follows: the 1 L of artificial saliva consisted of 520.2 mL of distilled water, 0.1 mL of micromineral solution (1 L of micromineral solution containing 132 g of CaCl_2_·2H_2_O, 100 g of MnCl_2_·4H_2_O, 10 g of CoCl_2_·6H_2_O and 80 g of FeCl_3_·6H_2_O), 208.1 mL of buffer solution (1 L of buffer solution containing 35 g of NaHCO_3_ and 4 g of NH_4_HCO_3_), 208.1 mL of macromineral solution (1 L of micromineral solution containing 5.7 g of Na_2_HPO_4_, 6.2g of KH_2_PO_4_, and 0.6 g of MgSO_4_), 1 mL of 0.1% resazurin solution, and 62.4 mL of reduction solution (added 570 mg of Na_2_S·7H_2_O to 100 mL of 0.04 N of NaOH). The artificial rumen fluid was prepared by mixing rumen fluid with artificial saliva at a ratio of 1:2.

According to the method of Menke and Steingass [13], 200 mg of substrate was accurately weighed and placed at the bottom of a glass gas-tight syringe (120 mL, Changzhou yangming glass production Co., Ltd., Changzhou, China), and 30 mL of artificial rumen fluid was injected, sealed with a deoxidizer, then placed into incubator shaker (IS-RDH1, Suzhou JieMEI Electronics Co., Ltd., Suzhou, China) and incubated at 39 °C. There was a total of 21 glass gas-tight syringes, including 6 repetitions per treatment and 3 repetitions in blank. The gas production was measured by directly observing the scale on the glass gas-tight syringe at 3, 5, 7, 10, 20, 22, and 24 h after incubation, and the net gas production value was calculated by subtracting the average of gas generated in the blank.

At 24 h of incubation time, fermentation was halted by placing the glass gas-tight syringe into an ice bath. Subsequently, the pH value was promptly measured using a pH meter (Sartorius AR company, Gottingen, Germany). The rumen digesta was stored at −80 °C for future DNA extraction. The rumen fluid was then filtered through 4 layers of gauze, and 1 mL of rumen fluid was mixed with 200 μL of 25% metaphosphoric acid. Next, through vortexing and shaking, the mixture underwent centrifugation at 10,000× *g* at 4 °C for 10 min. The supernatant was meticulously filtered using a 13 mm nylon syringe filter with a pore size of 0.45 μm for VFA analysis. The VFA concentrations, including acetic acid (AA), propionic acid (PA), isobutyric acid (isoBA), butyric acid (BA), isovaleric acid (isoVA), and valeric acid (VA), were determined by gas chromatography (CP-3800, Varian Medical Systems Company, Palo Alto, CA, USA) with a column size of 30 m × 0.32 mm × 0.15 μm (DB-FFAP, Onlysci, China), in triplicate. The GC condition was based on the method referred to by Yang et al. [14]; in brief, the column was initially heated to a temperature of 80 °C for a duration of 2 min. Subsequently, the temperature was raised to 150 °C at a controlled rate of 10 °C/min for an additional 2 min. Following this, the temperature was further increased to 180 °C at a rate of 15 °C/min, maintaining this temperature for 5 min. The entire process lasted for a total of 18 min. The carrier gas used was helium, maintained at a consistent flow rate of 3 mL/min.

### 2.4. Chemical Analysis

Approximately 1 g of DFPE was extracted using 10 mL of extraction reagent (1% HCl-Methanol) by vortexing for 1 min, in triplicate. The mixture was then placed in an ultrasonic ice-water bath for 30 min. Subsequently, the extracted solution underwent filtration using a 0.22 μm organic phase filter membrane and was subsequently preserved at −20 °C for future analysis. A total of 15 kinds of phenolics were determined. The standard phenolics, including gallic acid, chlorogenic acid, catechin, epicatechin, protocatechuic acid, caffeic acid, syringate, rutin, coumaric acid, naringin, vanillic acid, myricetin, luteolin, quercetin, and kaempferol, were obtained from Merck (Merck Co., Ltd., Darmstadt, Germany). The phenolic compounds of all extract solutions were determined using an Agilent 1260 HPLC (Agilent Technologies, Santa Clara, CA, USA) coupled to an Agilent 6420 triple quadrupole mass spectrometer with a column of ZORBAX Eclipse Plus C_18_ (3.5 µm, 2.1 × 150 mm) operated at 35 °C, in triplicates.

All the feed (substrate) samples were passed through a 1 mm screen for analysis of chemical composition, in triplicate. The dry matter (DM), ash, crude protein (CP), ether extract (EE), calcium (Ca), and phosphorus (P) were analyzed according to the method of AOAC [15], and the gross energy (GE) was determined by using a calorimeter (Parr 6200, Moline, IL, USA). The rumen fluid amino nitrogen (NH_3_-N) was analyzed according to the method of Tian et al. [16]. The acid detergent fiber (ADF) and neutral detergent fiber (NDF) were determined according to the method of Van Soect et al. [17].

### 2.5. DNA Extract and Detection

The DNeasy^®^ PowerSoil^®^ Pro Kit (QIAGEN, Hilden, Germany) was used to extract the total microbial DNA from 18 rumen fluid samples according to the kit instructions. The concentration and purity of extracted DNA were determined by a NanoDrop 2000 spectrophotometer (Thermo Fisher Scientific, Waltham, MA, USA).

Bacterial 16S rRNA gene fragments (V3–V4) were amplified from the extracted DNA using primers Ba9F (5′-GAGTTTGATCMTGGCTCAG-3′) and Ba515Rmod1R (5′-CCGCGGCKGCTGGCAC-3′). The PCR condition was based on an earlier method reported by Zhang et al. [18] with minor modifications. Briefly, the process begins with an initial denaturation at 95 °C for 3 min, followed by 27 cycles of denaturation at 95 °C for 30 s, annealing at 55 °C for 30 s, and elongation at 72 °C for 45 s, concluding with a final extension at 72 °C for 10 min.

The PCR reactions were conducted using the following components: 4 μL of 5× TransStart FastPfu buffer, 2 μL of 2.5 mM deoxynucleoside triphosphates (dNTPs), 0.8 μL of each primer (5 μM), 0.4 μL of TransStart FastPfu DNA Polymerase, and 10 ng of the extracted DNA. Subsequently, distilled deionized water (ddH_2_O) was added to bring the total volume to 20 μL. Next, agarose gel electrophoresis (2%) was carried out to confirm the amplicon’s size. Finally, the amplicons were subjected to paired-end sequencing on the Illumina MiSeq sequencing platform (using PE300) through Majorbio Bio-Pharm Technology Co., Ltd. in Shanghai, China.

The raw sequences underwent quality control using FASTP software (https://github.com/OpenGene/fastp, version 0.19.6, accessed on 18 January 2024) and were merged using FLASH software (https://ccb.jhu.edu/software/FLASH/index.shtml, version 1.2.11, accessed on 18 January 2024). Specifically, the reads are filtered based on quality scores, removing bases with quality scores < 20 at the tail end. A 50 bp window was applied, and if the average quality value within the window was <20, the back-end bases were truncated. Reads < 50 bp after quality control (QC) were excluded, and reads containing N bases were eliminated. Pairs of reads were merged into a single sequence based on the overlap relationship between PE reads, requiring a minimum overlap length of 10 bp. The maximum allowable mismatch ratio in the overlap region of the merged sequence was 0.2, and non-conforming sequences were discarded. Samples were distinguished by barcode and primers at the sequence’s start and end. The sequence orientation was adjusted accordingly.

The operational taxonomic unit (OTU) clustering was performed at 97% sequence identity using the UPARSE software (http://drive5.com/uparse/, version 11, accessed on 18 January 2024) and compared with the Silva 16S rRNA database (https://www.arb-silva.de/, version 138, accessed on 18 January 2024), with a comparison threshold of 70%.

### 2.6. Statistical Analysis

In this study, the effect of DFPE on gas production and rumen fermentation parameters was performed by SPSS 27 (Chicago, IL, USA) using one-way analysis of variance (ANOVA), and Tukey’s test was used to compare the differences among the 3 treatment groups. The effects of DFPE on ruminal bacteria parameters were investigated using nonparametric tests (Kruskal–Wallis test). The significance level was *p* < 0.05. The correlation between rumen fermentation parameters and bacterial communities was analyzed using the Spearman test.

## 3. Results

### 3.1. The Phenolic Composition of Dragon Fruit Peel Extract

The top five phenolics with the highest content in dragon fruit peel extract were naringin, caffeic acid, vanillic acid, quercetin, and protocatechuic acid, respectively. The bottom five phenolics with the lowest content were luteolin, catechin, gallic acid, epicatechin, and chlorogenic acid, respectively (Table 2).

### 3.2. DFPE on Gas Production and Rumen Fermentation

**Figure 1:**
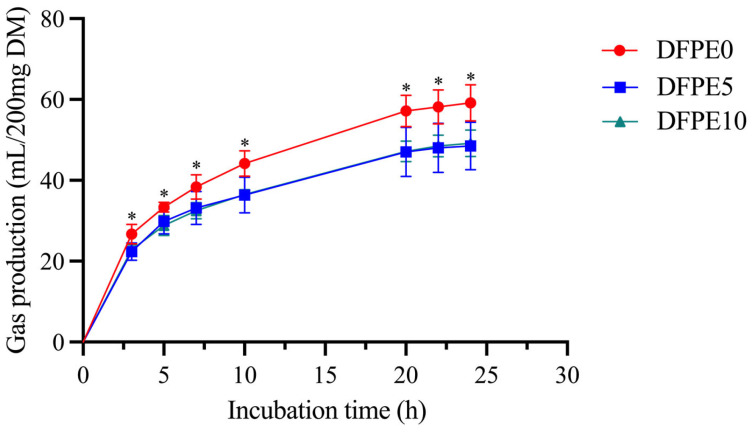
Effect of DFPE on gas production in vitro. DFPE0, base diet; DFPE5, base diet + 5 g/kg DFPE; DFPE10, base diet + 10 g/kg DFPE; DM, dry matter, “*” indicated significant difference.

The gas production curve tended to be flat after 24 h of incubation in all treatments, and the supplementation of DFPE significantly reduced the cumulative gas production at 24 h (Figure 1). No significant differences (*p* > 0.05) were observed in pH, propionic acid, isobutyric acid, butyric acid, and the ratio of acetic acid to propionic acid among all treatments. DFPE0 had a significantly lower (*p* < 0.05) ammonia nitrogen, total volatile fatty acid, isovaleric acid, and valeric acid, whereas DFPE0 showed a significantly higher (*p* < 0.01) acetic acid than the DFPE5 and DFPE10, see Table 3 below.

### 3.3. Rumen Bacterial Richness, Diversity, and Composition

**Figure 2:**
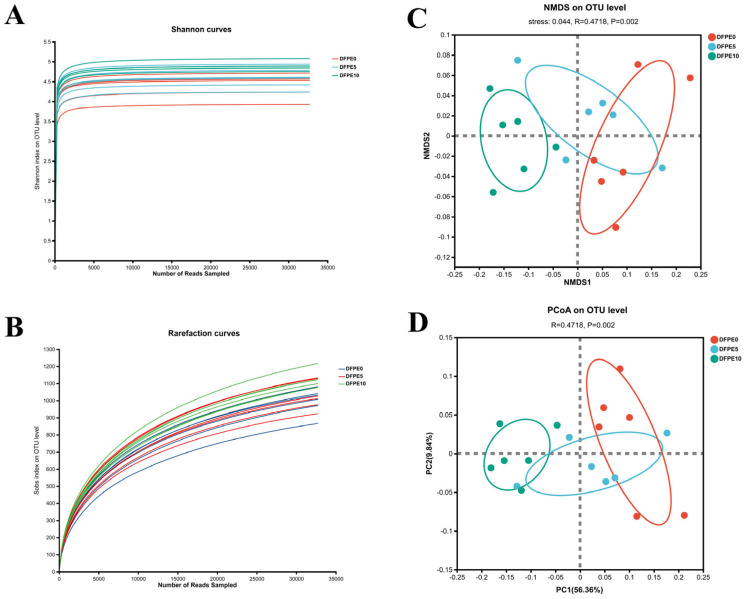
Effect of DFPE on ruminal bacterial diversity. (**A**) The Shannon curves. (**B**) The rarefaction curves. (**C**) NMDS analysis. (**D**) PCoA based on the Bray–Curtis distance.

After removing low-quality or chimeric sequences, there was a total of 1,081,480 high-quality sequences, and the number of total effective bases was 523,605,765 obtained from the 18 samples. A total of 2921 OTUs were identified and categorized into 15 phyla, 23 classes, 61 orders, 100 families, 230 genera, and 485 species. The rarefaction curve based on Sobs and Shannon indexes revealed that (Figure 2A,B) as the number of sequences increased, the curves gradually leveled off, signifying saturated coverage and sufficient sequencing depth in the present experiment.

As shown in Table 4, the coverage index for all samples was greater than 99%. The indexes of Sobs, Shannon, and Ace were higher (*p* < 0.05) in the DFPE10 than DFPE5 and DFPE0 groups. No difference (*p* > 0.05) was observed in the Chao index.

The non-metric multidimensional scaling (NMDS) revealed a microbial community and distinct structure. Based on the distance calculation of Bray–Curtis, the stress of NMDS at the OTU level was 0.044 (Figure 2C), indicating the criteria for obtaining a dependable representation of the alterations in the bacterial community structure. The PCoA noted based on Bray–Curtis, with the contribution values of principal component (PC) 1 and PC2 for sample differences, were 56.36% and 9.84%, respectively (Figure 2D ANOSIM; *p* = 0.002).

**Figure 3:**
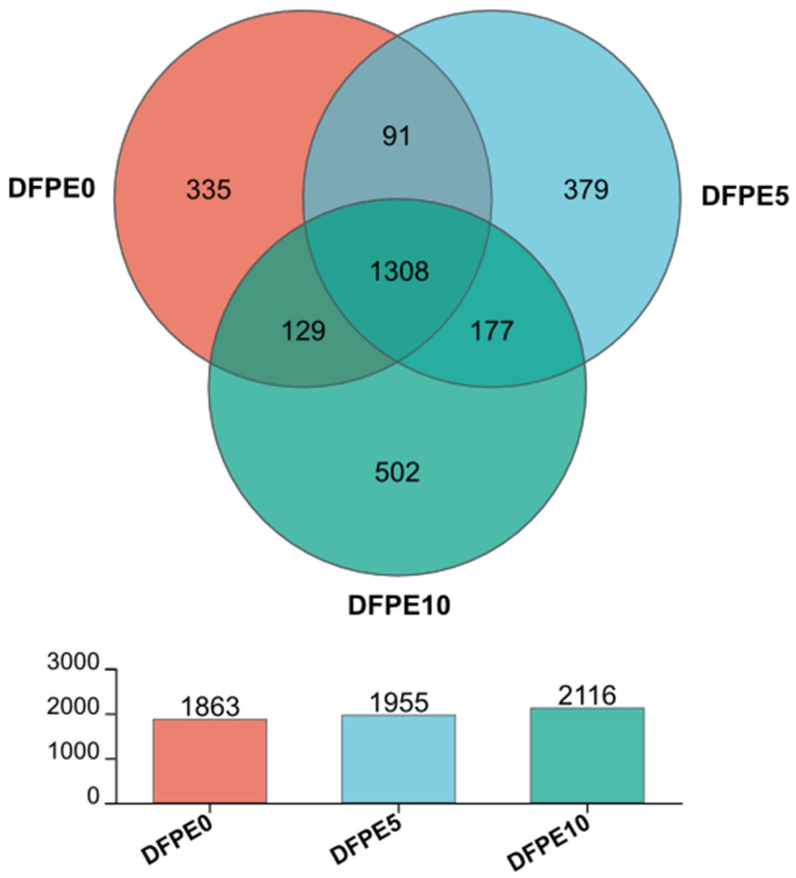
The Venn diagram.

In the Venn diagram shown (Figure 3), each ellipse symbolizes a group, and the intersecting region among the ellipses signifies the common OTU shared between the groups, with the numerical value indicating the quantity of OTUs. The DFPE0, DFPE5, and DFPE10 groups had 1863, 1955, and 2116 OTUs, respectively. A total of 1308 OTUs were observed across all groups, and specifically, 335, 379, and 502 OTUs were detected for the DFPE0, DFPE5, and DFPE10 groups, respectively.

### 3.4. Rumen Bacteria Composition at Phylum Level

**Figure 4:**
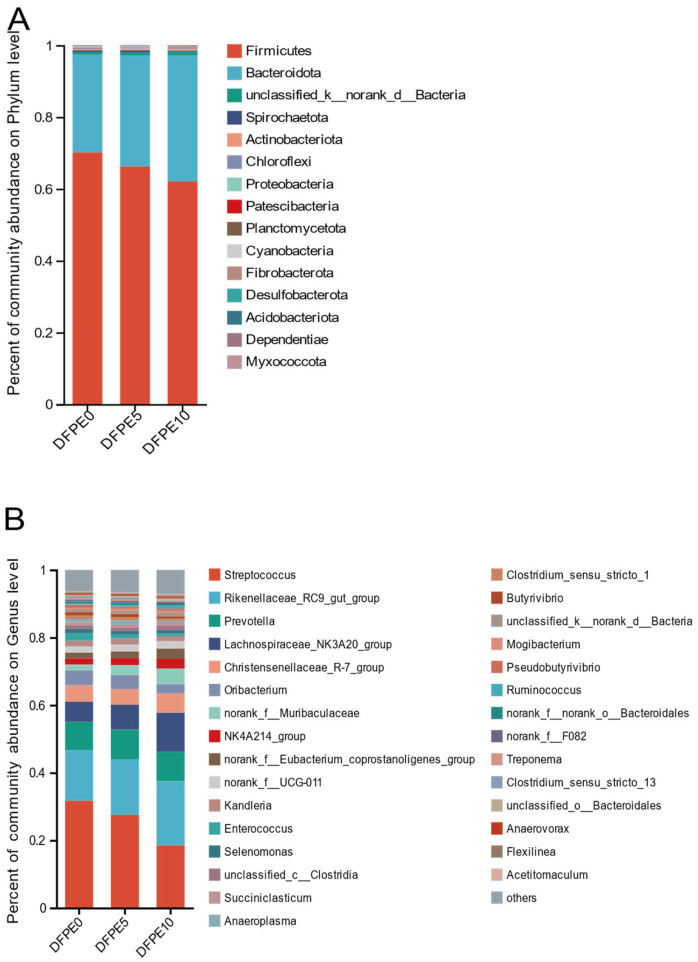
Effect of DFPE on the composition of rumen bacteria community. (**A**) Top 15 ruminal bacterial communities at the phylum level. (**B**) Top 30 ruminal bacterial communities at the genus level.

At the phylum level (Figure 4A and Table 5), we found that *Firmicutes* (DFPE0, DFPE5, and DFPE10 were 70.21%, 66.30%, and 62.13%, respectively) was the predominant bacteria phylum, followed by *Bacteroidota* (DFPE0, DFPE5, and DFPE10 were 27.21%, 30.84%, and 35.06%, respectively), and *Firmicutes* and *Bacteroidota* together accounted for more than 97% of the total bacterial abundance at the phylum level. Furthermore, followed by *unclassified_k_norank_bacteria* (DFPE0, DFPE5, and DFPE10 were 0.68%, 0.86%, and 1.01%, respectively), the ratio of *Firmicutes* to *Bacteroidetes* in DFPE0, DFPE5, and DFPE10 groups were 2.65, 2.21, and 1.79, respectively.

We employed the nonparametric Kruskal–Wallis test to assess the relative richness at the phylum level (at least one treatment was relative abundance > 0.5%) among the three groups. There were no differences (*p* > 0.05) between the DFPE0 and DFPE5 groups in the relative abundances of each bacterium at the phylum level. In comparison to the DFPE0 group, the DFPE10 group exhibited a significant rise in the relative abundances of the *Bacteroidota* and *unclassified_k_norank_bacteria* phyla, accompanied by a notable decrease (*p* < 0.05) in the relative abundance of the *Firmicutes* phylum, as well as a reduced ratio of *Firmicutes* to *Bacteroidota*. Furthermore, in contrast to the DFPE10 group, the DFPE5 group demonstrated an increase (*p* < 0.05) in the ratio of *Firmicutes* to *Bacteroidota*.

### 3.5. Rumen Bacteria Composition at Genus Level

A total of 181, 193, and 197 bacterial genera were observed in the DFPE0, DFPE5, and DFPE10 groups, respectively (Figure 4B). The top 30 relative abundances of bacteria at the genus level with a total of 17 genera were found with an abundance greater than 1% (Table 6). In the DFPE0 group, *Streptococcus* (31.71%) and *Rikenellaceae_RC9_gut_group* (14.93%) were the dominant genera, followed by *Prevotella* (8.40%), *Lachnospiraceae_NK3A20_group* (5.95%), *Christensenellaceae_R-7_group* (4.88%), and *Oribacterium* (4.45%). Combined, these taxa made up more than 70% of the overall bacterial composition. In the DFPE5, *Streptococcus* (27.5%) and *Rikenellaceae_RC9_gut_group* (16.52%) were predominant genera, followed by *Prevotella* (8.70%), *Lachnospiraceae_NK3A20_group* (7.35%), *Christensenellaceae_R-7_group* (4.69%), and *Oribacterium* (4.14%); collectively, these taxa comprised roughly 65% of the overall bacterial composition. In DFPE10, *Rikenellaceae_RC9_gut_group* (18.89%), *Streptococcus* (18.52%), and *Lachnospiraceae_NK3A20_group* (11.55%) were the predominant genera, followed by *Prevotella* (8.75%), *Christensenellaceae_R-7_group* (5.74%), and *norank_f_Muribaculaceae* (4.60%); collectively, these taxa comprised roughly 65% of the overall bacterial composition.

We employed the nonparametric Kruskal–Wallis test to assess the relative richness at the genus level (at least one treatment was relative abundance > 1%) among the three groups. The relative abundances of genera *Kandleria*, *Prevotella*, *Succiniclasticum*, *norank_f_UCG-011*, *Selenomonas*, and *Anaeroplasma* showed no significant differences (*p* > 0.05) among all the treatments. The relative abundances of *Lachnospiraceae_NK3A20_group* (*p* < 0.01), *Christensenellaceae_R-7_group* (*p* < 0.01), *norank_f_Eubacterium_coprostanoligenes_group* (*p* < 0.01), *unclassified_c_Clostridia*, and *unclassified_k_norank_d_Bacteria* (*p* = 0.01) were elevated in the DFPE10 group compared to the DFPE0 and DFPE5 groups, with no discernible differences (*p* > 0.05) between the DFPE0 and DFPE5 groups. Conversely, the relative abundances of *Streptococcus* (*p* < 0.01) and *Oribacterium* (*p* = 0.01) were lower in the DFPE10 group than in the DFPE0 and DFPE5 groups, with no significant distinctions observed between the DFPE0 and DFPE5 groups. The relative abundance of *Enterococcus* (*p* < 0.01) was decreased in the DFPE5 and DFPE10 groups. The relative abundances of *Rikenellaceae_RC9_gut_group* (*p* < 0.01), *norank_f_Muribaculaceae* (*p* = 0.01), *NK4A214_group* (*p* < 0.01) were lower in the DFPE10 group than in the DFPE0 group, and there was no significant difference in the DFPE5 group when compared to either the DFPE0 or DFPE10 groups.

### 3.6. Spearman Correlations between Bacterial Communities and Rumen Fermentation Parameters

**Figure 5:**
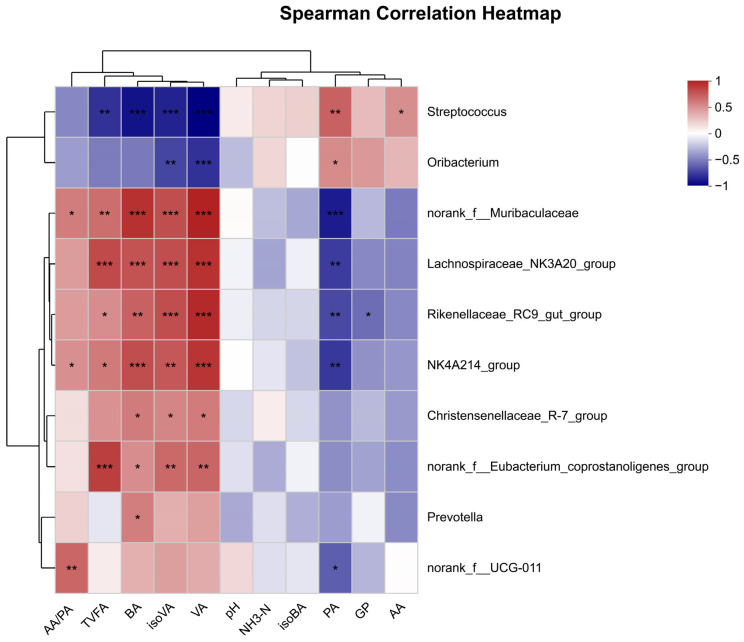
Spearman correlation analysis of the top 10 bacteria genera with gas production and ruminal fermentation parameters. (* *p* < 0.05, ** *p* < 0.01, *** *p* < 0.001).

The correlation between rumen fermentation parameters and bacterial communities is presented in Figure 5. The relative abundance of *Streptococcus* was negatively (*p* < 0.001, r < −0.70) correlated with TVFA, BA, isoVA, and VA; however, it showed a positive (*p* < 0.01, r = 0.66) correlation with PA. The relative abundances of *g__Rikenellaceae_RC9_gut_group*, *g__Lachnospiraceae_NK3A20_group*, *g__norank_f__Muribaculaceae*, and *g__NK4A214_group* were positively (*p* < 0.05, r > 0.66) correlated with BA, isoVA, and VA. However, it showed a negative (*p* < 0.05, r < 0.62) correlation with PA. The relative abundance of *g__norank_f__Eubacterium_coprostanoligenes_group* showed a positive (*p* < 0.01, r > 0.62) correlated with TVFA, isoVA, and VA. Additionally, the relative abundance of *g__Oribacterium* was negatively (*p* < 0.001, r < −0.71) correlated with VA. Moreover, TVFA had a positive (*p* < 0.001, r > 0.75) correlation with the relative abundances of *g__Lachnospiraceae_NK3A20_group*, and *g__norank_f__Eubacterium_coprostanoligenes_group*, whereas it showed a negative (*p* = 0.001, r < 0.70) correlation with the relative abundance of *g__Streptococcus*.

## 4. Discussion

### 4.1. Effect of DFPE on Gas Production and Rumen Fermentation

GP serves as a crucial indicator of carbohydrate digestion, exhibiting a negative correlation with NDF content and a positive correlation with starch levels [19]. An excess of GP can contribute to impaired ruminal function, particularly under highly concentrated feeding conditions, leading to the likelihood of cattle and sheep experiencing a subacute rumen bloat state [20]. Polyphenols demonstrate potential in preventing rumen bloat [21]. As anticipated, our finding suggests that DFPE has the ability to reduce GP in vitro. A previous study [22] has reviewed that these compounds possess the capability to reduce GP and CH_4_. Yu et al. [23] reported that naringin could reduce CH_4_ production in in vitro rumen fermentation. This reduction in GP may be attributed to the presence of phenolic compounds in DFPE, including naringin, caffeic acid, vanillic acid, and quercetin (Table 2), which may contribute to this reduction in GP. Furthermore, we observed that supplement levels did not influence GP. One possible reason may be that some gas-producing bacteria (e.g., methanogens) have varying degrees of sensitivity to DFPE, which necessitates further investigation in the future.

On the other hand, phenolic compounds can induce the precipitation of soluble proteins by forming hydrogen bonds [24]. The correlation of this phenomenon with alleviating tissue swelling has been substantiated [25]. A previous study demonstrated that 10 g/kg of polyphenolic extract from *Eucommia ulmoides* leaf reduced ruminal NH_3_-N levels from 175.6 to 129.6 mg/L [26]. Yu et al. [23] reported that naringin (20 mg/kg of diet) had decreased NH_3_-N from 13.05 mg/dL to 11.41 mg/dL in dairy cows. Conversely, the protein-binding property of phenolic compounds can mitigate ruminal fermentation, thereby enhancing nitrogen use efficiency [27]. In the present study, our observations indicate that DFPE reduced NH_3_-N levels, implying that DFPE has the ability to mitigate the degradation of dietary protein in the rumen and has the potential to enhance protein utilization.

Ruminal pH serves as a valuable indicator for assessing both ruminal health and optimal function. Consistent with the findings reported by Matra et al. [7], no significant differences in pH were observed in this study, suggesting that DFPE has the capability to maintain an appropriate acid–base status without inducing rumen acidosis. In contrast, the pH observed in this experiment ranges from 6.12 to 6.17, which differs from the optimal pH range (6.2–7.2) for microbial activity. As stated by Jackson et al. [28], this deviation may be attributed to constraints inherent in the in vitro fermentation system used in this experiment, wherein fermentation by-products such as VFA cannot be absorbed by the artificial rumen, leading to their accumulation and subsequent pH reduction. Nevertheless, the pH values remained within the normal range [29].

VFA are end-products of carbohydrates and provide the main source of energy for ruminant metabolism. Polyphenols have the potential to increase VFA by modifying the rumen bacteria population and increasing bacterial richness and diversity [30,31]. Gao et al. [32] reported that feeding beef bulls with polyphenol-rich red cabbage extract (120 g/d/head) resulted in a significant increase in total VFA from 83.06 mmol/L to 89.19 mmol/L. In the present study, we found that DFPE significantly increased total VFA. Similarly, previous studies [33,34] have reported that quercetin can increase ruminal VFA and decrease GP in vitro. In contrast, the present study revealed a comparatively lower total VFA content (10.78 to 11.95 mmol/L), while Sun et al. reported rumen total VFA ranges from 13.17 to 23.59 mmol/L (in vivo) [35]. This may be related to the lower microbial concentration in vitro than in vivo [36].

Butyric acid accounts for about 70% of the daily energy metabolism of ruminants [37]. Higher butyrate concentrations also enhance ruminal structure and function, enhance nutrient absorption, promote gut health, and improve health benefits for ruminants [38]. Remling et al. [39] noted that butyric acid also stimulated the growth of the ruminal papilla, thereby increasing the absorptive surface area of ruminants. In the present study, we found that DFPE had no effect on acetic acid, propionic acid, or butyric acid. A similar result was found by Silva et al. [40]. On the other hand, branched-chain VFA (BCVFA), such as isobutyric acid, isovaleric acid, and valeric acid, is utilized by ruminal microbes as a source of carbon skeleton for synthesizing branched-chain amino acids [41]. In the present study, we found higher concentrations of isovaleric acid and valeric acid from DFPE treatments. This indicates that DFPE has the potential to augment the population of cellulolytic bacteria and improve fiber digestibility.

### 4.2. Effect of DFPE on Bacteria Communities In Vitro

The well-being of the rumen is, in part, contingent upon the appropriate richness, diversity, and stability exhibited by the ruminal microbiome. The alpha diversity indexes of Sobs, Shannon, and Ace were increased by DFPE supplementation. In the present study, we examined the rumen microbiota using 16S rRNA sequencing analysis. As previously elucidated and documented in Sika deer [42], we observed that the predominant bacteria belonged to the phyla *Firmicutes* and *Bacteroidota*, with a high relative ratio of *Firmicutes* to *Bacteroidetes* in this study. This occurrence may be attributed to the fact that Sika deer typically inhabit cold and high-altitude regions [43], where a high ratio of *Firmicutes* to *Bacteroidetes* is advantageous for fat deposition, helping animals adapt to severe cold climates [44], thereby enhancing their resilience to these challenging conditions. Polyphenols are regarded as candidate compounds for prebiotics due to their interactions with gut microbiota [45]. The observations from the present study revealed that the relative abundance of *Bacteroidetes* increased with the inclusion of DFPE supplementation, while *Firmicutes* exhibited a significant decrease. In agreement with our study, Sutoyo et al. [46] reported that dietary intake of phenolic compounds is associated with a reduced proportion of *Firmicutes* and an increased proportion of *Bacteroidetes*.

Bacteroidetes are primarily involved with the degradation of carbohydrates, fats, and proteins. At the *Bacteroidetes* phylum, *Rikenellaceae_RC9_gut_group*, *Prevotella*, and *norank_f_Muribaculaceae* emerged as the predominant bacterial genera in our study, consistent with findings from a previous study by Si et al. [47]. The family *Rikenellaceae* participates in the degradation of structural carbohydrates, produces succinate and propionate as fermentation end products, and promotes lipid metabolism [48], while the family *Muribaculaceae* has been demonstrated to exhibit probiotic effects and is associated with the innate immune system and the absorption and utilization of fat [49]. A previous report indicated that the relative abundance of *Muribaculaceae* is negatively correlated with fat deposition and is affected by the digestion of carbohydrates [50]. In the present study, we observed an increase in the relative abundance of *Rikenellaceae_RC9_gut_group* and *norank*_*f_Muribaculaceae* with DFPE supplementation. Meanwhile, these bacterium abundances were positively correlated with butyric acid, isovaleric acid, and valeric acid. Similarly, a previous study showed that feeding Hu sheep with the polyphenol-rich fruit *Nitraria tangutorum* could increase the abundance of *Rikenellaceae_RC9_gut_group* and *Christensenellaceae_R-7_group* [51]. In this study, a positive correlation was identified between *Rikenellaceae_RC9_gut_group* and butyric acid content. Loubet Filho et al. [52] observed that cyanidin-3-glucoside led to an augmentation in the abundance of *Muribaculaceae*, suggesting that the bacteria of the *Muribaculaceae* family may be the target of cyanidin-3-glucoside, potentially contributing to the positive effects of cyanidin-3-glucoside on metabolism and inflammation.

Firmicutes exhibit the capability to break down fibers into short-chain fatty acids. Within the Firmicutes phylum, *Christensenellaceae_R-7_group* is commonly found in the intestinal tract and mucosa of both humans and animals, and it plays a crucial role in preserving the health of the host [53]. Previous studies indicated that *Christensenellaceae_R-7_group* has the potential to stimulate the development of the rumen, improving the absorption and digestion of nutrients. They have also exhibited a positive correlation with the breakdown of dietary protein, a negative correlation with diseases, such as inflammation [54,55,56], and a positive correlation with nipple width, epithelial thickness, and stratum corneum thickness [57]. Certainly, it is worth noting that microorganisms with different taxonomic features may serve the same function. Conversely, microorganisms with the same taxonomic characteristics may fulfill distinct functions. For example, *NK4A214_group* (*Oscillospiraceae* family) and *Christensenellaceae_R-7_group* (*Christensenellaceae* family), despite not belonging to the same family, may contribute individually or cooperatively to the promotion of ruminal biohydrogenation [58]. *Lachnospiraceae_NK3A20_group* (*Lachnospiraceae* family) plays an important role in rumen development and the promotion of rumen fermentation by increasing butyrate production [57]. *Oribacterium* belongs to the *Lachnospiraceae* family and has been recognized as one of the predominant bacteria in the rumen of cows that are fed forage-based diets [59], but its role in the rumen has not been reported yet. In the present study, we found that polyphenols from DFPE increased the abundance of *Lachnospiraceae_NK3A20_group*, *Christensenellaceae_R-7_group*, and *NK4A214_group*, indicating that polyphenols from DFPE can regulate rumen fermentation and potentially promote ruminal epithelial development, which may explain the rise in butyrate. A similar result was found in a report by Xu et al. [60]. Polyphenols could potentially play a crucial role in safeguarding microorganisms from oxidative stress [16]. This seems to explain the increased abundance of multiple bacteria caused by DFPE.

*Streptococcus* serves as the primary lactic acid fermenter in the rumen, converting starch and lactic acid into acetic acid and propionic acid. Since lactic acid is a stronger acid than VFA, the accumulation of lactate frequently causes a decrease in ruminal pH (5.0 or less) [61]. *Streptococcus* displays a notable tolerance to acidity, enabling them to thrive and multiply at low pH levels [62]. This characteristic may hinder the growth of other bacteria during such conditions [62]. On the other hand, the *Streptococcus* genus is recognized as one of the primary pathogens responsible for inducing mastitis [63]. In the present study, we reveal that DFPE had the ability to reduce the relative abundance of *Streptococcus*. The reason may be that polyphenols have the capability to engage with bacterial proteins, resulting in the inhibition of bacterial nucleic acid synthesis, the modulation of cell membrane function and fluidity, adjustments to cell wall integrity and synthesis, an impact on cell metabolism, and the prevention of biofilm formation [64]; in addition, polyphenols have the ability to increase the production of short-chain fatty acids and inhibit the growth of pathogenic bacteria [65]. Similarly, we also found *Streptococcus* had a negative correlation with the butyric acid content. *Enterococcus* is considered a probiotic, which contributes to the maintenance of lactic acid-utilizing bacteria activity, stimulates the growth of rumen microorganisms, and enhances the supply of gluco-propionic acid-producing energy for the host ruminants [66]. In the present study, we found that *Enterococcus* was decreased by DFPE. The mechanism for this is still unclear and requires future studies. A potential reason might be the increased relative abundance of certain microbes indirectly influencing the growth of other microbes [16].

## 5. Conclusions

The current study indicates that DFPE enhanced in vitro rumen fermentation serving as a potential ruminal modulator due to its ability to maintain ruminal pH, reduce gas production, decrease the concentration of NH_3_-N and acetic acid, and increase the concentrations of total VFA, isovaleric acid, and valeric acid. It also indicates that DFPE enhanced in regulation of rumen bacteria in Sika deer. However, further studies are needed to elucidate how DFPE enhances tumen fermentation in in vivo feeding trials.

## Figures and Tables

**Table 1:** The composition and nutrient level of the basal substrate (g/kg of DM) for in vitro fermentation.

Ingredients	Content
Napier grass	456.54
Peanut vine	43.46
Peanut shell	159.43
Garlic peel	106.34
Soybean waste	106.34
Soybean meal	37.06
Corn	72.73
Wheat bran	7.41
Calcium bicarbonate	2.59
Salt	5.34
Premix ^1^	2.76
Total	1000
Nutrient level	
Gross energy, MJ/kg of DM	13.06
Dry matter, g/kg	956.97
Ash	98.95
Crude protein	143.68
Ether extract	42.87
Neutral detergent fiber	363.08
Acid detergent fiber	281.47
Calcium	14.62
Phosphorus	3.26

^1^ 1 kg premix consists of vitamin premix 700,000 IU, Fe 10 g, Zn 3 g, Cu 0.75 g, Mn 3 g, Se 0.01 g, Co 0.02 g.

**Table 2:** Composition of phenolic compounds in DFPE (ng/g of DM).

Item	Mean	SD
Gallic acid	30.88	1.02
Chlorogenic acid	47.42	3.62
Catechin	27.88	2.99
Epicatechin	35.57	1.50
Protocatechuic acid	1363.07	109.56
Caffeic acid	2682.22	250.81
Syringate	199.99	51.93
Rutin	174.90	24.15
Coumaric acid	731.49	45.76
Naringin	9977.35	317.84
Vanillic acid	2649.25	206.65
Myricetin	314.84	19.93
Luteolin	10.94	1.62
Quercetin	2187.91	133.37
Kaempferol	49.68	2.74

DFPE, dragon fruit peel polyphenolic extract; SD, standard deviation; values represent the mean of three replicates.

**Table 3:** Effect of DFPE on gas production and rumen fermentation.

Item	DFPE0	DFPE5	DFPE10	SEM	*p*-Value
GP_24h_, mL/g of DM	295.83 a	242.50 b	245.83 b	7.85	<0.01
pH	6.17	6.12	6.15	0.02	0.53
NH_3_-N, mg/dL	34.80 a	30.90 b	31.57 b	0.44	<0.01
TVFA, mmol/L	10.78 b	11.83 a	11.95 a	0.20	0.02
Molar proportion (mmol/100 mmoL)					
AA	58.88 a	57.69 b	57.48 b	0.22	<0.01
PA	25.22	24.85	24.04	0.26	0.17
isoBA	0.61	0.71	0.65	0.03	0.49
BA	11.82	12.04	12.56	0.15	0.09
isoVA	0.73 b	0.94 a	1.02 a	0.04	<0.01
VA	2.75 b	3.77 a	4.25 a	0.19	<0.01
AA/PA	2.34	2.33	2.39	0.03	0.58

SEM, standard error of the mean; GP, gas production, g/mL of DM; NH_3_-N, ammonia nitrogen; TVFA, total volatile fatty acid; AA, acetic acid; PA, propionic acid; isoBA, isobutyric acid; BA, butyric acid; isoVA, isovaleric acid; VA, valeric acid; AA/PA, the ratio of acetic acid to propionic acid. Different letters indicated significant difference.

**Table 4:** Analysis of alpha diversity index in the present study.

Item	DFPE0	DFPE5	DFPE10	SEM	*p*-Value
Sobs	999.67 b	1033.17 b	1118.50 a	19.95	0.03
Shannon	4.43 b	4.59 b	4.89 a	0.07	0.01
Ace	1218.43 b	1228.20 b	1340.36 a	0.01	0.04
Chao	1183.92	1189.80	1287.96	22.59	0.07

Different letters indicated significant difference.

**Table 5:** Effect of DFPE on ruminal microbiota at the phylum level (relative abundance > 0.5).

Item	DFPE0	DFPE5	DFPE10	SEM	*p*-Value
*Firmicutes*	70.21 b	66.3 ab	62.13 a	1.195	<0.01
*Bacteroidota*	27.21 b	30.84 ab	35.06 a	1.176	0.01
*unclassified_k_norank_d_Bacteria*	0.68 b	0.86 ab	1.01 a	0.043	<0.01
*Firmicutes*: *Bacteroidota*	2.65 b	2.21 ab	1.79 a	0.130	0.01

Different letters indicated significant difference.

**Table 6:** Effect of DFPE on ruminal microbiota at the genus level (relative abundance in at least one treatment > 1%).

Genus	DFPE0	DFPE5	DFPE10	SEM	*p*-Value
*Rikenellaceae_RC9_gut_group*	14.93 b	16.52 ab	18.89 a	0.57	<0.01
*Prevotella*	8.40	8.70	8.75	0.21	0.58
*norank_f_Muribaculaceae*	1.60 b	2.90 ab	4.60 a	0.44	0.01
*Streptococcus*	31.71 b	27.50 b	18.52 a	1.71	<0.01
*Lachnospiraceae_NK3A20_group*	5.95 b	7.35 b	11.55 a	0.73	<0.01
*Christensenellaceae_R-7_group*	4.88 b	4.69 b	5.74 a	0.15	<0.01
*norank_f_Eubacterium_coprostanoligenes_group*	1.73 b	1.96 b	3.02 a	0.16	<0.01
*NK4A214_group*	1.88 b	2.09 ab	2.94 a	0.14	<0.01
*Oribacterium*	4.45 b	4.14 b	2.72 a	0.25	0.01
*norank_f_UCG-011*	1.83	2.00	2.16	0.08	0.29
*Kandleria*	1.73	1.89	1.53	0.11	0.44
*unclassified_c_Clostridia*	0.95 b	1.03 b	1.22 a	0.04	0.01
*Succiniclasticum*	1.02	0.90	1.02	0.03	0.05
*Selenomonas*	1.29	0.99	0.98	0.10	0.68
*Enterococcus*	2.17 b	1.18 a	0.77 a	0.18	<0.01
*Anaeroplasma*	0.98	1.24	0.57	0.12	0.05
*unclassified_k_norank_d_Bacteria*	0.68 b	0.86 b	1.01 a	0.04	<0.01

Different letters indicated significant difference.

## Data Availability

Raw data associated with each sample have been submitted to the National Center for Biotechnology Information under study accession number PRJNA1083787.
